# Influence of N- and/or P-restriction on bone metabolism in young goats

**DOI:** 10.1017/S0007114524002150

**Published:** 2024-10-14

**Authors:** Luisa S. Zillinger, Annette Liesegang, Karin Hustedt, Nadine Schnepel, Helga Sauerwein, Marion Schmicke, Cornelia Schwennen, Alexandra S. Muscher-Banse

**Affiliations:** 1 Institute for Physiology and Cell Biology, University of Veterinary Medicine Hannover, Foundation, Hannover 30173, Germany; 2 Institute of Animal Nutrition and Dietetics, Vetsuisse Faculty Zurich, University of Zurich, Zurich 8057, Switzerland; 3 Center for Applied Biotechnology and Molecular Medicine (CABMM), Zurich, Switzerland; 4 Institute of Animal Science, Physiology Unit, University of Bonn, Bonn 53115, Germany; 5 Clinic for Diseases of Cattle, University of Veterinary Medicine Hannover Foundation, Hannover 30173, Germany; 6 Institute for Animal Nutrition, University of Veterinary Medicine Hannover Foundation, Hannover 30173, Germany

**Keywords:** Bone metabolism, Dietary nitrogen and phosphate, Fibroblast growth factor 23, Goat

## Abstract

Ruminants can recycle nitrogen (N) and phosphorus (P), which are essential for vital body processes. Reduced N- and P-intake in ruminants is desirable for economic and ecologic reasons. Simultaneous modulation of mineral homoeostasis and bone metabolism occurs in young goats. This study aimed to investigate potential effects of dietary N- and/or P-restriction on molecular changes in bone metabolism. The twenty-eight young male goats were fed a control diet, an N-reduced diet, a P-reduced diet or a combined N- and P-reduced diet for 6–8 weeks. The N-restricted goats had lower plasma Ca concentration and higher plasma osteocalcin (OC) and CrossLaps concentrations. The P-restricted goats had reduced plasma inorganic phosphate (P_i_) concentrations and increased plasma Ca concentrations. Due to the initiation of a signalling pathway that inhibits the fibroblast growth factor 23 (FGF23) expression, this was lower with P-restriction. Consequently, lower P_i_ concentrations were the main factor influencing the reduction in FGF23. The changes in mineral homoeostasis associated with P-restriction led to a reduction in OC, bone mineral content and mineral density. Simultaneously, bone resorption potentially increased with P-restriction as indicated by an increased receptor activator of NF-κB ligand/osteoprotegerin (OPG) ratio and an increase in OPG mRNA expression. Additionally, the increased mRNA expression of the calcitonin receptor during P-restriction points to a higher number of osteoclasts. This study demonstrates an impairment of bone remodelling processes in young goats by N- or P-restriction. With P-restriction, bone mineralisation rate was potentially reduced and bone quality impaired, while with N-restriction, bone remodelling increased.

To protect the environment and its resources, reducing the nitrogen (N) and phosphorus (P) content in the diet of ruminants is a much-discussed option. Ruminants can compensate for reduced dietary N-intake quite well due to the ruminohepatic cycle that allows the use of urea^(1–3)^. Ruminants are also able to maintain endogenous phosphate (P_i_) homoeostasis during P-restriction because they can recycle P_i_^(4,5)^. N and P are both essential for the survival of the micro-organisms in the rumen of the host animal and must therefore be available in sufficient quantities. N is an important source of protein synthesis in ruminants, and P_i_ is essential for energy metabolism, cell signalling and bone mineralisation^(6–8)^. Despite the ability of ruminants to compensate for reduced N- and P-intake, previous studies have shown that severe changes in mineral homoeostasis occurred. N-restriction resulted in lower blood concentration of Ca and calcitriol, the active form of vitamin D^(9,10)^, while P-restriction resulted in increased blood concentration of Ca with concomitantly reduced blood P_i_ concentration^(11,12)^. Most of the body’s Ca and P_i_ are stored in the bones, where they form the two main components of the bone mineral hydroxyapatite, which strengthens the bone matrix^(6)^. Changes in the Ca and P_i_ concentration in the blood are balanced by the interaction of various hormones that regulate reabsorption and excretion in the intestine and kidney. Additionally, these changes in concentration are regulated by a release of Ca and P_i_ from bone and are stored in the bone matrix. The bone-derived hormone fibroblast growth factor 23 (FGF23) is the most important regulator of P_i_ homoeostasis in monogastric species^(13,14)^ and ruminants^(15)^. Extracellular P_i_ acts as a signalling molecule on the bone and directly regulates the release of FGF23 from the bone^(16)^. It is assumed that P_i_ sensing in bone is mediated by the two Na-dependent P_i_ transporters 1 and 2 (PiT1, SLC20A1 and PiT2, SLC20A2)^(17)^. While PiT2 is essential for the regulation of P_i_-dependent FGF23 secretion in mice^(18)^, PiT1 is essential for bone mineralisation in rats^(19)^. In monogastric species, low levels of P_i_ in the blood reduce the excretion of FGF23. Consequently, the expression of renal Na-dependent P_i_ transporters remains unchanged at lower FGF23 levels, allowing the reabsorption of P_i_ via the kidneys^(20)^. Apart from P_i_, the synthesis of FGF23 in bone is also regulated by other factors. Phosphate-regulating endopeptidase X-linked (PHEX) that is expressed in osteoblasts and osteocytes is necessary for an increase in FGF23 gene transcription^(21)^. Hence, a reduction of PHEX is expected with the down-regulation of FGF23. Another inhibitory pathway of FGF23 secretion is induced by insulin and insulin-like growth factor 1 (IGF1) through binding of the insulin receptor (INSR) and the insulin-like growth factor 1 receptor (IGF1R). This activates the phosphatidylinositol-3-kinase (PI3K)/serine/threonine kinase (Akt) pathway. Its activation inhibits Forkhead box 1 (FOXO1), which leads to the inhibition of FGF23^(22)^. An FGF23-inducing pathway in bone is that of Janus kinase 2 (JAK2)/signal transducer and activator of transcription 3 (STAT3) signalling^(23)^. This pathway is activated by leptin and growth hormone (GH) when binding to their receptors’ leptin receptor (LEPR)^(24)^ and growth hormone receptor (GHR), respectively^(25,26)^. In addition to FGF23, mineral homoeostasis is also regulated by parathyroid hormone (PTH) which is secreted by the parathyroid gland^(27)^. Once secreted from the parathyroid gland, PTH binds to its receptor parathyroid hormone 1 receptor (PTH1R) on osteoblasts regulating bone resorption^(28)^. By binding to PTH1R, PTH induces bone resorption through stimulation of receptor activator of NF-κB ligand (RANKL)^(29)^. Simultaneously, osteoprotegerin (OPG) is inhibited by PTH, resulting in the release of Ca and P_i_ from bone^(29)^. OPG acts as a decoy receptor of RANKL because it binds RANKL and therefore blocks the binding of RANKL to its receptor activator of NF-κB (RANK)^(30)^. For storing Ca and P_i_ in bone, osteoblasts are activated initiating bone formation^(31,32)^. Osteoblastic maturation is induced by runt-related transcription factor 2 (RUNX2), alkaline phosphatase (ALP) and osteocalcin (OC), among others^(31,33)^. From previous studies on ruminants, it is known that changes in mineral homoeostasis due to reduced N- and P-intake impaired bone metabolism^(15,34)^. As a next step, the changes which are caused in the bone by a reduction of dietary N and/or P will be characterised molecularly.

P-restriction is expected to result in reduced FGF23 expression, and thus it is hypothesised that there is an increase in the inhibitory PI3K/Akt pathway with a simultaneous reduction in the stimulatory JAK2/STAT3 pathway. N-restriction disrupted the somatotropic axis in goats with lower IGF1 concentrations^(35)^. Therefore, an increase in FGF23 was hypothesised in the goats in this study due to reduced PI3K/Akt activation. Finally, the bone mineral density and the bone mineral content will be examined to see whether these postulated molecular changes also influence the bone macroscopically.

## Material and methods

### Animals and feeding regimens

Information about the animals and the feeding regimen has already been published in a previous study^(36)^. To avoid unnecessary repetition of animal model experiments, organs not used in the study of Weber et al.^(36)^ were taken in this study. Twenty-eight male Coloured German Goats from a commercial goat farm received (1) a control diet (16·48 % crude protein (CP), 0·48 % P, 1·30 % Ca), (2) an N-reduced diet (8·35 % CP, 0·51 % P, 1·20 % Ca), (3) a P-reduced diet (16·86 % CP, 0·11 % P, 1·20 % Ca) or (4) an N- and P-reduced diet (8·10 % CP, 0·11 % P, 1·20 % Ca) for six to eight weeks. At the beginning of the experiment, the animals had an initial body weight of 19·04 (sd 2·2) at an age of 10 weeks^(36)^. The pellets were produced by a specialised feed manufacturer for animal research feed production (ssniff Spezialdiäten GmbH). The diets had approximately 12·7 MJ ME/kg DM and were isoenergetic. For further details on the ingredients and chemical composition of the diets, see Table 1 and Weber et al.^(36)^.

**Table 1.** Components and composition of wheat straw and pelleted concentrate diets*

Items	Wheat straw	N+/P+	N-/P+	N+/P-	N-/P-
Components (as fed basis) (g/kg)					
Soyabean meal		68·0	57·0	68·0	57·0
Urea		24·5	–	24·5	–
Wheat starch		378·0	385·0	379·0	390·0
Beet pulp		399·0	415·0	399·0	410·0
Mineral–vitamin premix†		10·0	10·0	10·0	10·0
MgHPO_4_. 3 H_2_O		9·3	9·3	–	–
MgO		–	–	2·2	2·2
NaH_2_PO_4_. 2 H_2_O		9·7	10·0	0·4	0·9
NaCl		1·4	1·2	1·4	1·2
NaHCO_3_		–	–	5·0	5·0
CaCO_3_		14·3	14·2	14·3	14·2
Sipernat 22S‡		41·8	58·1	52·3	69·5
Molasses		10·0	10·0	10·0	10·0
Soyabean oil		34·0	30·0	34·0	30·0
Composition§					
DM (g/kg)	904·0	880·0	874·0	884·0	875·0
Nutrients (g/kg DM)					
Crude ash	39·8	100·0	112·0	101·0	115·0
CP	25·4	165·0	83·5	169·0	81·1
ADFom	579·0	87·5	96·1	87·1	84·6
aNDFom	855·0	157·0	169·0	179·0	173·0
Crude fat	24·3	43·2	49·2	47·5	43·4
Urea	BDL	27·8	BDL	31·1	BDL
Ca	2·8	12·6	12·5	12·3	11·8
P	0·6	4·8	5·1	1·1	1·1
Na	0·2	3·4	3·7	3·5	3·7
Mg	0·3	2·6	2·7	2·7	2·6
K	15·0	8·2	8·2	8·7	8·1
Cl	3·8	2·0	2·2	2·3	2·1
S	0·9	2·1	2·2	2·3	2·1
Vitamin D_3_ (IU/kg DM)	BDL	1000	1144	1075	1051
ME (MJ/kg DM)	8·3	12·8	12·4	12·9	12·5
DCAD|| (meq/kg DM)	230	167·6	171·7	166·5	178·4

N+/P+, control diet; N-/P+, N-restricted diet; N+/P-, P-restricted diet; N-/P-, N- and P-restricted diet; ADFom, acid-detergent fibre expressed exclusive of residual ash; aNDFom, neutral-detergent fibre assayed with heat stable amylase expressed exclusive of residual ash; CP, crude protein; BDL, below detection level; ME, metabolisable energy; DCAD, dietary cation–anion difference.

* Parts of the data already published in Weber et al.^(36)^.

† Mineral–vitamin premix per kg: 0·2 g P; 12·1 g Ca; 1·7 g Na; 2·2 g Mg; 1 200 000 IU vitamin A; 120 000 IU vitamin D; 10 000 mg vitamin E; 675 mg vitamin K; 4960 mg iron; 6336 mg Zn; 501 mg Cu; 3000 mg Mn; 201 mg Co; 15 mg Se; 202 mg I.

‡ Sipernat 22S (Evonik Industries AG) – a fine particle silica that can be used as highly absorbent carrier substance, flow regulator, anti-caking and anti-dusting agent in the food and feed industry.

§ Composition analysed by the Association of German Agricultural Investigation and Research Centre (VDLUFA).

|| DCAD (meq/kg DM) = (meq Na + meq K) - (meq Cl + meq S).

Seven goats per feeding group were housed together and water was available *ad libitum*. Each animal was fed 55 g/kg^0·75^ pelleted concentrate per animal twice a day. Additionally, the goats received 25 % of the concentrate weight as wheat straw. To ensure documentation of the average intake of nutrients for each animal, the goats received their diet individually and the amount eaten was documented daily. The body weight of the goats was measured once a week.

### Body fluid and tissue sampling

Blood samples were taken from the *vena jugularis* with lithium heparinate-coated syringes and serum syringes (Sarstedt) shortly before euthanasia using a standard abattoir captive bolt stunning procedure (Annex IV Directive 2010/63/EU). Blood samples were always taken at the same time in the morning from the fasting animal to avoid diurnal effects. Plasma and serum samples were centrifuged (2000 *g* at room temperature, 15 min) for separation and stored at −20°C until further analysis.

Two goats per d were killed due to technical reasons (Ussing chamber experiments). Animals from two feeding groups were slaughtered alternately each day to avoid significant time effects.

Shortly after euthanasia, a metacarpal bone and a rib were dissected. For this purpose, the flesh was removed from the bones, then immediately frozen in liquid N_2_ and stored at −80°C until further preparation. The metacarpal bone was examined using µCT, and the mineral content was then analysed. The rib bone was separated into cortical and medullary sections, and the cortical portion was used for quantitative PCR (qPCR) analysis.

### Biochemical and immunological assays

To determine the N, P_i_ and Ca status of the animals, blood plasma concentrations of urea (commercial kit, R-Biopharm AG), P_i_ and Ca were measured colorimetrically with standard spectrometric methods^(37,38)^ (interassay CV 3·79 % (urea), 1·88 % (Ca) and 5·05 % (P_i_); intra-assay CV 1·2 % (urea), 2·85 % (Ca) and 1·42 % (P_i_)). Plasma concentration of bone-specific alkaline phosphatase was measured by a competitive ELISA (Quidel Corporation). Plasma OC and CrossLaps (CTX) were also measured by competitive ELISA (TECOmedical Group and Immundiagnostik AG). The serum concentrations of leptin were measured with a bovine-specific competitive enzyme immunoassay^(39)^. Plasma GH and insulin as well as serum IGF1 were measured in the Clinical-Endocrinological Laboratory at the University of Veterinary Medicine Hannover, Foundation. Plasma GH was measured using an in-house ELISA. Plasma insulin and serum IGF1 were measured using RIA (Beckman Coulter GmbH)^(40)^.

### Determination of mineral content and density, ash, Ca, P, Mg and Zn content of the metacarpus

Mineral content and density of the metacarpus were measured by quantitative computer tomography (Stratec XCT bone scanner, Stratec Medizinaltechnik GmbH). Total distal and trabecular content and density were measured at 10 % of the total length of the metatarsi. To determine the total medial content and density as well as cortical medial content and density, the bones were scanned at 50 % of the total length, in the middle of the diaphysis. To determine the mineral content, the bones were sawn into small pieces. Subsequently, the content of almost fat-free DM of the bones was determined by lypophilisation for about 48 h, followed by defatting with petroleum ether by using the SOXTHERM (C. Gerhardt GmbH & Co. KG) and drying at 80°C. According to Rieger et al.^(41)^ and in accordance with the official methods of the VDLUFA (Association of German Agricultural Analytic and Research Institutes), the bones were then ground in a hammer mill and analysed for crude ash, crude fat (to determine fat-free DM), Ca, P, Mg and Zn.

### Quantitative reverse transcription real-time PCR

Total RNA from bone rib tissue was isolated using the RNeasy Fibrous Tissue Mini-Kit (Qiagen GmbH) following the manufacturer’s protocol. The RNA concentrations were measured spectrophotometrically using NanoDrop One (Thermo Fisher Scientific Inc. Waltham). The quality and integrity of the RNA were assessed using an RNA 6000 nanoassay for an Agilent 2100 Bioanalyzer (Agilent Technologies Deutschland GmbH). The RNA integrity number of all bone rib tissue samples was on average 8·69 (sem 0·12). Reverse transcription of 200 ng of isolated RNA for real-time qPCR was performed using random hexamers, oligo-dT primers and TaqMan™ Reverse Transcription Reagent (Thermo Fisher Scientific Inc.) by the manufacturer’s protocol.

To determine the mRNA abundance of Akt1, Akt2, ALP, calcitonin receptor (CALCR), FGF23, FOXO1, GHR, IGF1R, INSR, JAK2, LEPR, OC, OPG, PHEX, PiT1, PiT2, PTH1R, RANK, RANKL, RUNX2, STAT3 and vitamin D receptor (VDR), SYBR Green® PCR assays with specific primers (Table 2) were used. Reaction mixtures of 20 µl contained SensiFAST^TM^ SYBR No-Rox Mix (BioCat GmbH), 200 nmol/l of specific primers and 16 ng of reverse-transcribed complementary DNA. For amplification (3 min at 95°C; 40 cycles of 10s at 95°C and 30s at 60°C) and detection of the PCR products, a real-time PCR cycler (CFX96^TM^, Bio-Rad Laboratories GmbH) was used. To determine the melting curve, the thermal profile began with an incubation period of 10 min at 55°C with a gradual increase in temperature (0·5°C per 10s) up to 95°C. To evaluate the rRNA expression of 18S in bone rib tissue as a reference gene, caprine gene-specific TaqMan primers and probes were synthesised by TIB Molbiol Syntheselabor GmbH (Table 3). The reaction mixture of 20 µl each contained TaqMan™ Gene Expression Master Mix (Thermo Fisher Scientific Inc.), 16 ng of reverse transcript complementary DNA, 300 nmol/l of specific primers and 100 nmol/l of specific probe. PCR products were amplified (50°C, 2 min; 95°C, 10 min; 40 cycles of 95°C, 15 s and 60°C, 1 min) and analysed on a real-time PCR cycler (CFX96^TM^, Bio-Rad Laboratories GmbH). Absolute copy numbers were determined using calibration curves generated with cloned PCR fragment standards^(50)^. The specificity of amplicons was verified by sequencing (Microsynth Seqlab GmbH) and using NCBI Blast (http://blast.ncbi.nlm.nih.gov/Blast.cgi). The expressions of the reference genes ribosomal protein L19 (*RPL19*) and ribosomal protein S9 (*RPS9*) were quantified with SYBR Green® PCR assays (Table 2). The best consideration as a reference gene for normalisation was 18S rRNA, calculated by NormFinder software (https://www.moma.dk/normfinder-software). Reactions were performed twice and included water as a no-template control.

**Table 2.** Primers used for SYBR Green assays in compact bone rib tissue from young goats

Gene*	Primers and Probes (5’ → 3’)	Accession number	References
Akt1	Forward: TTCAAGCCTCAGGTCACGTC Reverse: ATGCTGTCGTCTTGGTCAGG	NM_001285750·1	This study
Akt2	Forward: GAACACCAGGCACCCGTTC Reverse: GAGACGATCTCTGCGCCATAA	XM_018062407·1 to XM_018062410·1	This study
ALP	Forward: CTGACCACTGGCAGCCCA Reverse: CAGGGTCTGCTGAGCTTGG	XM_018055210·1, XM_018055220·1, XM_018055232·1 and XM_018055238·1	^(15)^
CALCR	Forward: CCTGAATCGCCCACTTCCGG Reverse: AGCCATCCCAGGTGCGGTTG	XM_018047421·1	^(42)^
FGF23	Forward: TCCATGGAGACGGGCACATA Reverse: TGTGAAGTCCATGCAGAGGT	XM_018049064·1	^(15)^
FOXO1	Forward: TGTTTATCGAGCGCTTGGACT Reverse: TACTTTTAAGTGTAGCCTGCTCGC	XM_018056686·1	This study
GHR	Forward: CGTCTCTGCTGGTGAAAACA Reverse: GGATGTCGGCATGAATCTCC	NM_001285648·1	^(43)^
IGF1R	Forward: GGAGAATAATCCAGTCCTAGCACC Reverse: GAAGACTCCATCCTTGAGGGACTC	XM_018065947·1	^(35)^
INSR	Forward: TATGGGACTGGGGCAAACAC Reverse: TTTCACAGGATGCCTGGTCC	XM_018051134·1 to XM_018051137·1	^(44)^
JAK2	Forward: GTGCTGGCCGGCATAATCTA Reverse: ATTCCTTGTCGCCAGATCCC	XM_018052022·1	^(44)^
LEPR	Forward: TGAAACCACTGCCTCCATCC Reverse: CACTGGGAGACTGGCAGATTT	XM_018045220·1, XM_018045222·1 and XM_018045226·1	This study
OC	Forward: GTGATGCAGAGTCAGGCAAA Reverse: GTTGAGCTCACACACCTCCC	XM_018046289·1	^(15)^
OPG	Forward: GCAGTACGTCAAGCAGGAGT Reverse: CTGTATTTCGCTCCGGGGTT	XM_005689133·3	^(15)^
PHEX	Forward: TCCTGCAAACCCGCAAGTAT Reverse: TTGGTTGGTGGATGCACTGT	XM_018044036·1	^(15)^
PiT1	Forward: ATTCATCCTCCGTAAGGCAG Reverse: CAGCAATGGTGCTCCAGTGTACA	XM_018055327·1	^(35)^
PiT2	Forward: CCAATCTCGGGGACTCACTG Reverse: GGAACGGGGTCCTCCTTTTT	XM_018041866·1 to XM_018041870·1 and XM_018041872·1	^(45)^
PTH1R	Forward: ATCAACATCGTGCGGGTTCT Reverse: AGCCTGTACCTCACCATTGC	XM_018066729·1 to XM_018066731·1 and XM_018066733·1 to XM_018066735·1	^(46)^
RANK	Forward: GCCTCAGGCAGTGTGACT Reverse: TCCTGCGAGTTCTGACTGAC	XM_018039366·1	^(15)^
RANKL	Forward: AAAGCCATGGTGGAAGGTTC Reverse: TTGGAGATCTTGGCCCAACC	XM_018056784·1	^(15)^
RPL19	Forward: AGCCTGTGACTGTCCATTCC Reverse: ACGTTACCTTCTCAGGCATT	XM_005693740·3	^(47)^
RPS9	Forward: CGCCTCGACCAAGAGCTGAAG Reverse: CTCCAGACCTCACGTTTGTTCC	XM_018063497·1 and XM_018063498·1	^(48)^
RUNX2	Forward: CGCACCATGGTGGAGATCAT Reverse: AGGGCTACCACCTTGAAG	XM_018038794·1 to XM_018038798·1	^(15)^
STAT3	Forward: GACCTGGAGACGCACTCATT Reverse: CACTTGATCCCACGTTCCGA	NM_001314278·1	^(44)^
VDR	Forward: GCACTTCCTTACCTGACCCC Reverse: CCGCTTGAGGATCATCTCCC	XM_018047872·1 and XM_018047873·1	^(46)^

* Akt1, Akt serine/threonine kinase 1; Akt2, Akt serine/threonine kinase 2; ALP, alkaline phosphatase; CALCR, calcitonin receptor; FGF23, fibroblast growth factor 23; FOXO1, Forkhead box O1; GHR, growth hormone receptor; IGF1R, insulin-like growth factor 1 receptor; INSR, insulin receptor; JAK2, Janus kinase 2; LEPR, leptin receptor; OC, osteocalcin; OPG, osteoprotegerin; PHEX, phosphate-regulating endopeptidase X-linked; PiT1, pituitary-specific transcription factor 1; PiT2, pituitary-specific transcription factor 2; PTH1R, parathyroid hormone 1 receptor; RANK, receptor activator of NF-κB; RANKL, receptor activator of NF-κB ligand; RPL19, ribosomal protein L19; RPS9, ribosomal protein S9; RUNX2, runt-related transcription factor 2; STAT3, signal transducer and activator of transcription 3; VDR, vitamin D receptor.

**Table 3.** Primers and probes used for TaqMan^TM^ assays in bone rib tissue from young goats

Gene*	Primers and probes (5’ → 3’)	Accession number	References
18S rRNA	Forward: AAAAATAACAATACAGGACTCTTTCG Reverse: GCTATTGGAGCTGGAATTACCG FAM-TGGAATGAGTCCACTTTAAATCCTTCCGC-BBQ	AM711869·1	^(49)^

* 18S rRNA, 18S ribosomal RNA.

### Statistical analysis

Sample size (*n* 7/group) was determined based on metabolic data from a previous study^(10)^ with a statistical power of 0·8 and *α* error of 0·05. All data are given as means with their standard errors of the mean (sem) unless otherwise stated. GraphPad Prism version 9.3 (GraphPad Software) was used for data analysis.

All data were tested for normal distribution using Kolmogorov–Smirnov test. The results confirmed that the parameters were normally distributed, so the data are represented as measured means and sem. A two-way ANOVA was conducted to evaluate the main effects of N-intake and P-intake. *Post hoc* tests were not performed due to the absence of significant interactions between N-intake and P-intake factors.

A significance level of *P* < 0·05 was considered statistically significant, while *P* < 0·1 was used to define trends.

Potential linear relationships between mRNA expression levels and the correlation between gene expression with blood parameters were calculated using a simple correlation analysis with Pearson’s correlation coefficient. Potential outliers were tested with the ROUT method.

## Results

### Feed intake and growth performance

All animals were clinically healthy throughout the study. The clinical health of the animals was ensured through monitoring and care by a qualified veterinarian who examined the animals daily and closely observed their behaviour and physical condition. The data on the goats’ feed intake, daily weight gain and body weight have already been published by Weber et al.^(36)^.

### Blood metabolites

Blood metabolites are shown in Table 4. Some of the blood metabolites (Ca, GH, IGF1, P_i_ and urea) have already been published in Weber et al.^(36)^ and Zillinger et al.^(51)^. The plasma concentration of CTX was significantly increased in the animals receiving the N-restricted diet compared with the control group. Blood plasma concentration of OC was significantly lower in the P-restricted feeding groups and increased in the N-restricted animals compared with the control group. The blood plasma concentration of bone-specific alkaline phosphatase increased significantly with P-restriction compared with the control group. The OC concentration in plasma correlated negatively with the blood concentration of Ca (*r* = –0·62; *P* < 0·001) and positively with the blood concentration of P_i_ (*r* = 0·72; *P* < 0·001) and IGF1 (*r* = 0·58; *P* = 0·002). The blood concentrations of total protein, insulin and leptin were not affected by the different diets.

**Table 4.** Blood metabolites and hormones of young goats fed an N- and/or P-reduced diet (Mean values with their standard errors of the mean (SEM); seven animals per group)

Treatment	BALP (U/I)		CTX (ng/ml)		Leptin (ng/ml)		Insulin (µIU/ml)		OC (ng/ml)		Protein (g/l)	
	Mean	sem	Mean	sem	Mean	sem	Mean	sem	Mean	sem	Mean	sem
Control N	261·0	24·3	0·96	0·06	5·46	0·34	17·87	2·44	147·7	20·9	57·1	1·75
Reduced N	296·1	32·6	1·52	0·06	4·92	0·20	13·17	1·96	191·9	32·9*	52·8	1·57
Control P	226·7	19·8	1·21	0·09	5·26	0·34	15·59	2·16	244·6	26·9	55·0	1·55
Reduced P	330·5	29·9	1·27	0·10	5·12	0·23	15·45	2·44	98·7	5·34	54·9	1·95
*P*-value (N-reduction)	0·345		< 0·001		0·524		0·160		0·044		0·091	
*P*-value (P-reduction)	0·009		0·550		0·411		0·966		< 0·0001		0·940	

BALP, bone-specific alkaline phosphatase; CTX, CrossLaps; OC, osteocalcin; N, nitrogen; P, phosphorus.

For all parameters, *P*-value of NxP ≥ 0·112.

* *n* 6; due to the outlier test performed.

### Mineral content and density, ash, Ca, P, Mg and Zn content of the metacarpus

Results of the measurements by computer tomography are summarised in Table 5. The results of the mineral analysis of the metacarpal bone are summarised in Table 6. Total distal content and density, trabecular content and density as well as total medial content were significantly lower with P-restriction. Total medial density and cortical medial density and content were not affected by dietary modulation. The ash and Mg content were significantly lower with P-restriction, while the P content of the bone was reduced by trend with P-restriction. The Mg content increased by trend (*P* = 0·091) with N-restriction. No significant effects were observed for Zn with either N-reduction or P-reduction. However, an interaction between N and P was detected for Zn (data not shown; *P* = 0·049), whereas all other interactions had *P*-values ≥ 0·359.

**Table 5.** Metacarpal mineral content (mg/cm) and density (mg/cm^3^) of young goats fed an N- and/or P-reduced diet (Mean values with their standard errors of the mean (SEM); seven animals per group)

Treatment	Total distal content (mg/cm)		Total distal density (mg/cm^3^)		Trabecular content (mg/cm)		Trabecular density (mg/cm^3^)		Total medial content (mg/cm)		Total medial density (mg/cm^3^)		Cortical medial content (mg/cm)		Cortical medial density (mg/cm^3^)	
	Mean	sem	Mean	sem	Mean	sem	Mean	sem	Mean	sem	Mean	sem	Mean	sem	Mean	sem
Control N	130·7	7·02	327·0	12·6	57·00	3·90	316·8	16·6	93·38	2·99	520·1	13·5	90·90	2·86	835·2	10·2
Reduced N	121·3	4·13	324·0	6·94	50·62	2·64	298·6	10·3	90·81	2·26	528·7	15·5	88·87	2·25	828·5	9·69
Control P	135·2	5·99	342·1	10·7	58·69	3·49	329·7	14·9	95·55	1·57	535·6	14·2	92·86	1·55	842·8	8·97
Reduced P	116·7	4·54	309·0	7·12	48·91	2·80	285·7	10·0	88·65	3·16	513·3	14·3	86·91	3·11	820·9	10·0
*P*-value (N-reduction)	0·231		0·826		0·168		0·330		0·485		0·683		0·575		0·629	
*P*-value (P-reduction)	0·023		0·020		0·039		0·024		0·068		0·297		0·109		0·126	

N, nitrogen; P, phosphorus.

For all parameters, *P*-value of NxP ≥ 0·466.

**Table 6.** Effects of an N- and/or P-reduced diet on ash, Ca, P, Mg and Zn of the metacarpus of young goats (g/kg fat-free DM; mean values with their standard errors of the mean (SEM); seven animals per group)

Treatment	Ash (g/kg)		Ca (g/kg)		P (g/kg)		Mg (g/kg)		Zn (mg/kg)	
	Mean	sem	Mean	sem	Mean	sem	Mean	sem	Mean	sem
Control N	525·4	8·47	186·1	2·11	90·86	1·49	3·10	0·12	100·1	3·47
Reduced N	525·6	6·75	188·4	3·62	90·84	1·79	3·27	0·11	100·8	3·24
Control P	538·5	3·73	187·5	2·37	92·94	1·42	3·53	0·07	97·54	3·38
Reduced P	512·6	8·81	187·1	3·48	88·77	1·66	2·84	0·08	103·4	3·14
*P*-value (N-reduction)	0·983		0·594		0·992		0·091		0·873	
*P*-value (P-reduction)	0·016		0·928		0·074		< 0·001		0·200	

### Effects of a nitrogen- and/or phosphorus-reduced diet on mRNA expression of selected target genes in the rib bones

**Fig. 1.**
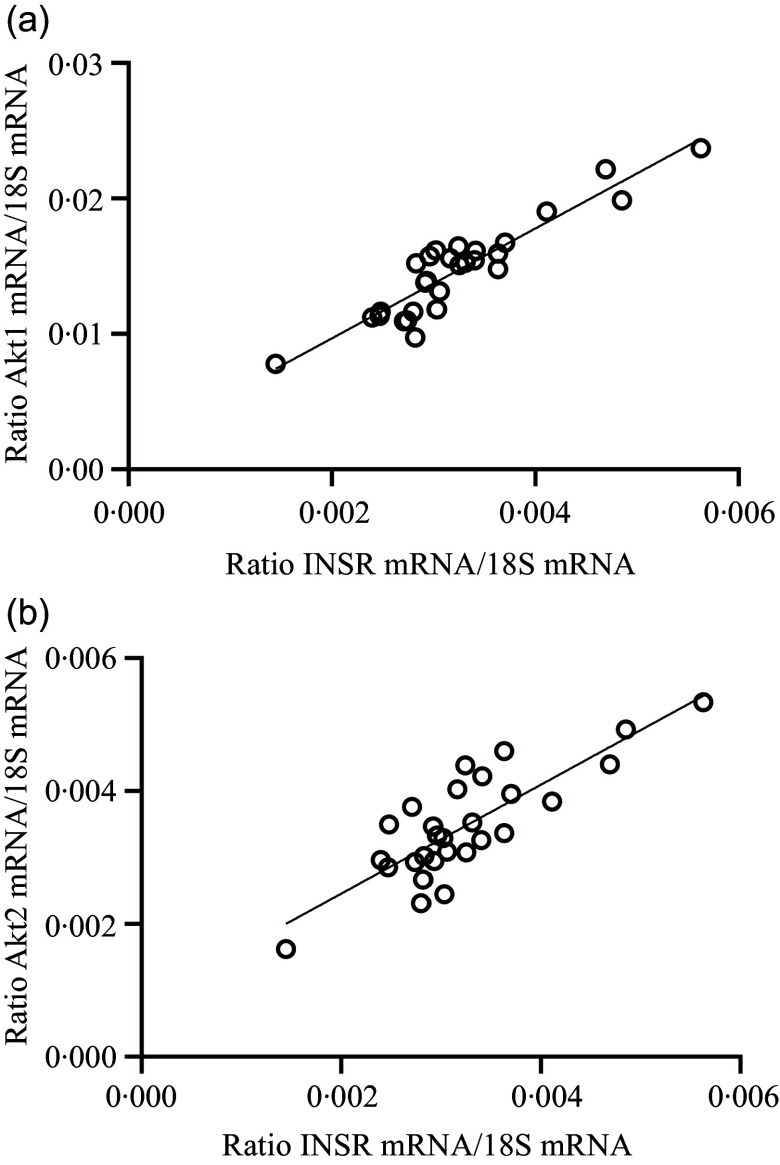
Linear relationship between insulin receptor (INSR) mRNA abundance and (a) Akt serine/threonine kinase 1 (Akt1) mRNA abundance in the bone cortex (*r* = 0·92, *P* < 0·001) and (b) Akt serine/threonine kinase 2 (Akt2) mRNA abundance in the bone cortex (*r* = 0·82, *P* < 0·001). The level of significance with Pearson’s correlation coefficient was set at *P* = 0·05.

**Fig. 2.**
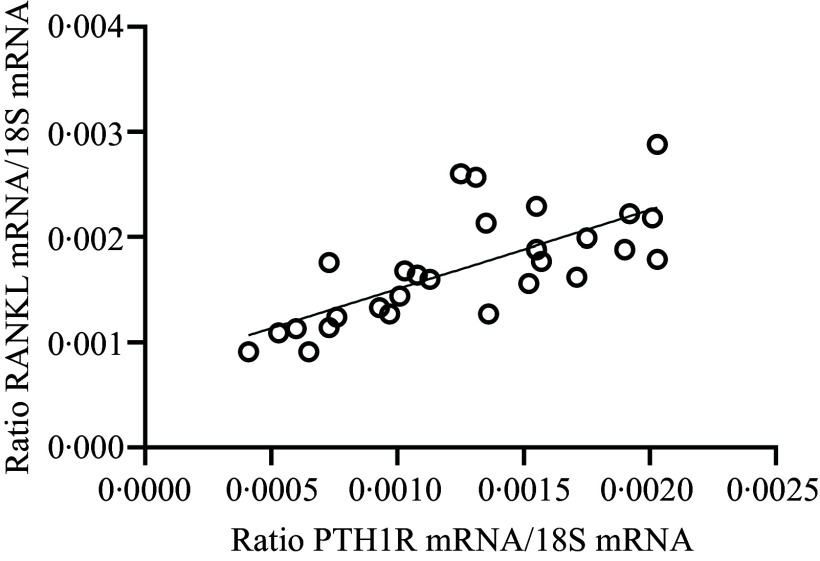
Linear relationship between mRNA abundance of parathyroid hormone 1 receptor (PTH1R) and mRNA abundance of receptor activator of NF-κB ligand (RANKL) in the bone cortex (*r* = 0·71, *P* < 0·001). The level of significance with Pearson’s correlation coefficient was set at *P* = 0·05.

The results of the mRNA expression of this study are shown in Table 7. The gene expression of ALP, PHEX, PiT2, RANK and VDR remained unchanged in all feeding groups. A significant increase in the mRNA expression of Akt1, Akt2, CALCR, GHR, IGF1R, INSR, JAK2, LEPR, PiT1, PTH1R and STAT3 was demonstrated in P-restricted feeding compared with the control animals. RUNX2 showed a strong tendency towards increased gene expression with P-restricted feeding (*P* = 0·052) as well as with simultaneous N- and P-restricted feeding (*P* = 0·075). A significant increase in mRNA expression of Akt1, FOXO1, OC, RANKL and STAT3 was also detected with N-restriction. The ratio of RANKL to OPG increased significantly with P-restriction, with an interaction also observed (data not shown; *P* = 0·049). For all other interaction parameters, *P*-value of NxP ≥ 0·075. The gene expression of INSR (*P* = 0·052) and PTH1R (*P* = 0·061) showed a tendency towards increased gene expression with N-restriction. INSR gene expression correlated positively with Akt1 (*r* = 0·92; *P* < 0·001; Fig. 1(a)) and Akt2 gene expression (*r* = 0·82; *P* < 0·001; Fig. 1(b)). PTH1R mRNA expression correlated positively with plasma CTX (*r* = 0·45, *P* = 0·016) and RANKL gene expression (*r* = 0·52; *P* = 0·004; Fig. 2). P-restricted feeding significantly lowered the mRNA expression of FGF23, OC and OPG. OC mRNA correlated negatively with plasma Ca and positively with plasma P_i_ (*r* = –0·49, *P* = 0·008 and *r* = 0·48, *P* = 0·010). FGF23 mRNA expression correlated positively with plasma P_i_ (*r* = 0·81; *P* < 0·001) and negatively with PTH1R mRNA (*r* = –0·37; *P* = 0·05).

**Table 7.** Relative mRNA expression levels (normalised to 18S rRNA) of various genes in compact bone rib tissue of young goats fed an N- and/or P-reduced diet (Mean values with their standard errors of the mean (SEM); seven animals per group)

Treatment	Akt1		Akt2		ALP		CALCR		FGF23		FOXO1	
	Mean	sem	Mean	sem	Mean	sem	Mean	sem	Mean	sem	Mean	sem
Control N	13·34 × 10^−3^	9·57 × 10^−4^	32·65 × 10^−4^	2·24 × 10^−4^	24·26 × 10^−3^	3·47 × 10^−3^	43·71 × 10^−5^	7·19 × 10^−5^	39·43 × 10^−5^	1·26 × 10^−4^	50·45 × 10^−4^	3·68 × 10^−4^
Reduced N	16·04 × 10^−3^	8·63 × 10^−4^	36·75 × 10^−4^	2·09 × 10^−4^	30·59 × 10^−3^	2·55 × 10^−3^	51·38 × 10^−5^	7·39 × 10^−5^	38·71 × 10^−5^	1·03 × 10^−4^	67·54 × 10^−4^	4·00 × 10^−4^
Control P	13·28 × 10^−3^	6·44 × 10^−4^	30·23 × 10^−4^	1·60 × 10^−4^	24·50 × 10^−3^	2·65 × 10^−3^	35·31 × 10^−5^	4·29 × 10^−5^	71·86 × 10^−5^	0·98 × 10^−4^	56·46 × 10^−4^	3·79 × 10^−4^
Reduced P	16·10 × 10^−3^	11·05 × 10^−4^	39·17 × 10^−4^	2·09 × 10^−4^	30·35 × 10^−3^	3·43 × 10^−3^	59·78 × 10^−5^	8·19 × 10^−5^	6·39 × 10^−5^	0·18 × 10^−4^	61·54 × 10^−4^	5·04 × 10^−4^
*P*-value (N-reduction)	0·035		0·129		0·148		0·423		0·946		0·005	
*P*-value (P-reduction)	0·029		0·002		0·180		0·016		< 0·001		0·363	

Treatment	GHR		IGF1R		INSR		JAK2		LEPR		OC	
	Mean	sem	Mean	sem	Mean	sem	Mean	sem	Mean	sem	Mean	sem
Control N	20·78 × 10^−4^	2·25 × 10^−4^	14·20 × 10^−4^	1·03 × 10^−4^	29·71 × 10^−4^	2·15 × 10^−4^	23·46 × 10^−3^	2·57 × 10^−3^	24·98 × 10^−4^	2·61 × 10^−4^	1·41·	0·16
Reduced N	24·14 × 10^−4^	1·81 × 10^−4^	16·06 × 10^−4^	1·30 × 10^−4^	35·03 × 10^−4^	2·09 × 10^−4^	24·93 × 10^−3^	2·42 × 10^−3^	30·54 × 10^−4^	3·15 × 10^−4^	2·14·	0·25
Control P	18·84 × 10^−4^	1·54 × 10^−4^	12·95 × 10^−4^	1·12 × 10^−4^	28·18 × 10^−4^	1·34 × 10^−4^	19·13 × 10^−3^	1·87 × 10^−3^	22·56 × 10^−4^	2·52 × 10^−4^	2·22·	0·24
Reduced P	26·09 × 10^−4^	2·09 × 10^−4^	17·31 × 10^−4^	0·94 × 10^−4^	36·56 × 10^−4^	2·36 × 10^−4^	29·26 × 10^−3^	2·26 × 10^−3^	32·96 × 10^−4^	2·72 × 10^−4^	1·33·	0·14
*P*-value (N-reduction)	0·196		0·214		0·052		0·626		0·145		0·006	
*P*-value (P-reduction)	0·008		0·006		0·004		0·002		0·010		0·0009	

Treatment	OPG		PHEX		PiT1		PiT2		PTH1R			
	Mean	sem	Mean	sem	Mean	sem	Mean	sem	Mean	sem		
Control N	8·93 × 10^−5^	1·56 × 10^−5^	27·90 × 10^−4^	2·34 × 10^−4^	15·63 × 10^−5^	1·22 × 10^−5^	13·53 × 10^−4^	1·98 × 10^−4^	11·16 × 10^−4^	1·38 × 10^−4^		
Reduced N	9·43 × 10^−5^	0·91 × 10^−5^	32·56 × 10^−4^	3·23 × 10^−4^	16·94 × 10^−5^	1·64 × 10^−5^	16·21 × 10^−4^	1·20 × 10^−4^	14·10 × 10^−4^	1·18 × 10^−4^		
Control P	11·29 × 10^−5^	1·48 × 10^−5^	33·79 × 10^−4^	3·36 × 10^−4^	13·63 × 10^−5^	1·07 × 10^−5^	15·32 × 10^−4^	1·34 × 10^−4^	10·15 × 10^−4^	1·14 × 10^−4^		
Reduced P	7·07 × 10^−5^	0·61 × 10^−5^	26·67 × 10^−4^	1·88 × 10^−4^	18·94 × 10^−5^	1·42 × 10^−5^	14·41 × 10^−4^	1·95 × 10^−4^	15·11 × 10^−4^	1·18 × 10^−4^		
*P*-value (N-reduction)	0·763		0·234		0·453		0·257		0·061			
*P*-value (P-reduction)	0·017		0·074		0·005		0·698		0·003			

Treatment	RANKL		RANK		RANKL/OPG ratio		RUNX2		STAT3		VDR	
	Mean	sem	Mean	sem	Mean	sem	Mean	sem	Mean	sem	Mean	sem
Control N	14·18 × 10^−4^	0·92 × 10^−4^	19·84 × 10^−4^	1·41 × 10^−4^	21·85·	3·87	46·52 × 10^−6^	4·54 × 10^−6^	20·67 × 10^−4^	1·76 × 10^−4^	16·68 × 10^−4^	1·89 × 10^−4^
Reduced N	19·94 × 10^−4^	1·36 × 10^−4^	22·96 × 10^−4^	1·63 × 10^−4^	22·48·	1·77	53·47 × 10^−6^	3·37 × 10^−6^	26·18 × 10^−4^	1·83 × 10^−4^	20·41 × 10^−4^	1·53 × 10^−4^
Control P	16·14 × 10^−4^	1·44 × 10^−4^	19·74 × 10^−4^	1·44 × 10^−4^	16·66·	1·94	44·76 × 10^−6^	4·08 × 10^−6^	20·11 × 10^−4^	1·40 × 10^−4^	16·60 × 10^−4^	1·41 × 10^−4^
Reduced P	17·99 × 10^−4^	1·34 × 10^−4^	23·06 × 10^−4^	1·58 × 10^−4^	27·67·	3·11	55·24 × 10^−6^	3·60 × 10^−6^	26·75 × 10^−4^	1·99 × 10^−4^	20·49 × 10^−4^	1·97 × 10^−4^
*P*-value (N-reduction)	0·001		0·151		0·858		0·188		0·023		0·128	
*P*-value (P-reduction)	0·258		0·127		0·005		0·052		0·007		0·113	

18S rRNA, 18S ribosomal RNA; N, nitrogen; P, phosphorus; Akt1, Akt serine/threonine kinase 1; Akt2, Akt serine/threonine kinase 2; ALP, alkaline phosphatase; CALCR, calcitonin receptor; FGF23, fibroblast growth factor 23; FOXO1, Forkhead box O1; GHR, growth hormone receptor; IGF1R, insulin-like growth factor 1 receptor; INSR, insulin receptor; JAK2, Janus kinase 2; LEPR, leptin receptor; OC, osteocalcin; OPG, osteoprotegerin; PHEX, phosphate-regulating endopeptidase X-linked; PiT1, pituitary-specific transcription factor 1; PiT2, pituitary-specific transcription factor 2; PTH1R, parathyroid hormone 1 receptor; RANK, receptor activator of NF-κB; RANKL, receptor activator of NF-κB ligand; RUNX2, runt-related transcription factor 2; STAT3, signal transducer and activator of transcription 3; VDR, vitamin D receptor.

## Discussion

In this study, the influence of N- and/or P-restriction on bone metabolism in young goats focuses on molecular-level changes. Our findings support previous research highlighting the critical role of P in bone mineralisation and structural integrity. The reduction in ash content observed with P-restriction underscores the importance of P-containing diets in maintaining hydroxyapatite crystals in bone. Concomitantly, the decrease in Mg content observed suggests a potential secondary effect, as Mg plays an important role in bone health, affecting both bone density and quality. The tendency for decreased bone P content under P-restriction emphasises the direct influence of P-containing diets on bone P levels, which is critical for the maintenance of hydroxyapatite crystals in bone. Interestingly, the tendency for increased Mg levels with N-restriction (*P* = 0·091) suggests a compensatory mechanism. N is essential for amino acid and protein synthesis, which are essential for bone matrix formation. N-restriction could potentially alter bone metabolism and lead to a compensatory increase in Mg uptake or retention as the body attempts to maintain bone homoeostasis under suboptimal nutrient conditions.

Furthermore, in our study, we observed that P-restriction not only affects bone mineralisation but also has significant effects on Ca homoeostasis. P is crucial for maintaining the balance of minerals in the body, and its restriction can lead to compensatory mechanisms that alter Ca metabolism. Specifically, P-restriction can lead to an increase in blood Ca levels as the body mobilises Ca from bones to maintain necessary P_i_ concentrations in the blood. This mobilisation process can potentially lead to an imbalance in mineral homoeostasis, which contributes to milk fever, especially in dairy cows. Milk fever, or hypocalcemia, is a metabolic disorder that often occurs in dairy cows around the time of calving and is characterised by low blood Ca levels. The disorder can be exacerbated by an imbalance of P and Ca in the diet. According to Keanthao et al.^(52)^, P-restriction leads to a reduction in bone P content and can cause a compensatory increase in blood Ca levels. This imbalance highlights the need for carefully balanced diets to prevent metabolic disorders such as milk fever.

In monogastric species, the reduced plasma concentration of P_i_ led to increased bone resorption by osteoclasts to release P_i_ from bone while inhibiting bone mineralisation by osteoblasts^(53,54)^. In rats fed a low energy diet, the concentration of bone formation marker OC in the serum, which represents late osteoblastic maturation^(55)^, reduced with a simultaneous reduction in the blood concentration of P_i_^(56)^. In the young goats of the current study, the lower OC concentration and the associated mRNA abundance during P-reduction can be explained by the fact that bone formation and thus OC concentration are energy-dependent, as in monogastric animals^(56)^. The positive correlation of plasma P_i_ with plasma OC and OC mRNA supports this assumption. An additional reason for the diminished OC gene expression could be a change in vitamin D metabolism during P-reduction, as the expression of the vitamin D activator, CYP27B1, in the kidney was reduced^(51)^. OC synthesis is triggered by calcitriol, the active hormonal form of vitamin D, via the VDR and a specific vitamin D-responsive element in the promoter of the OC gene^(57)^. In mice treated with calcitriol, this stimulated OC RNA abundance after binding to VDR because one of the effects of calcitriol on bone is to promote osteoblast differentiation^(58)^.

Furthermore, it is assumed that the high Ca concentrations in the blood during a P-reduction lower OC levels because in humans with hypercalcaemia, the blood concentration of OC was significantly reduced and therefore reduced bone formation was hypothesised^(59)^. This assumption is supported by the negative correlation of OC gene expression and OC in blood with Ca in blood.

An earlier study in N-restricted goats demonstrated increased bone turnover with an increase in the bone formation marker OC and a simultaneous increase in bone resorption marker CTX^(34)^. In the study by Elfers et al.^(34)^, the N-restricted goats developed reduced levels of plasma Ca that were thought to have caused the increase in CTX. At the same time, the blood calcidiol concentration increased, and it was hypothesised that this was the reason for the increase in OC concentration^(34)^, as a vitamin D dependency of OC was suspected in human bone cells^(60,61)^. In the present project, the blood concentration of calcidiol did not change, indicating that OC levels in goats are probably not influenced by calcidiol. Rather it appears that the reduction in blood Ca concentration alone caused the increase in bone turnover, which is reflected in an increase in both CTX and OC. The lower Ca concentration in the blood induces the secretion of PTH from the parathyroid glands in monogastric species^(62)^ as well as cows^(63)^. Unfortunately, no specific assay for measuring PTH in goat blood is commercially available. By tending to increase PTH1R expression in bone during N-restriction in this study, PTH could induce bone formation by osteoblasts, which in turn would stimulate bone resorption by osteoclasts^(64,65)^, to release increased Ca from the bone.

Despite the reduction in OC, it was hypothesised that early osteoblastic maturation increased in the P-restricted goats due to the trend towards increased RUNX2 gene expression. In a study on P-restricted mice, RUNX2 mRNA also increased, while OC mRNA expression was lower. It was hypothesised that P-restriction prevented differentiation from immature to mature osteoblasts^(31)^. The increase in the enzyme bone-specific alkaline phosphatase, which also functions as a bone formation marker^(55)^, during P-restriction presumably occurred to generate free P_i_ which can be provided to the bone cells in times of reduced blood P_i_ concentration^(66)^. A reduced bone mineralisation rate was also reflected in a lower bone mineral density and bone mineral content in the P-restricted goats as it was also seen in hens and pigs receiving a P-restricted diet^(67,68)^.

The observed increase in blood Ca concentration with P-restriction may be responsible for the increase in CALCR mRNA expression, as a study with CALCR knockout mice demonstrated that the presence of CALCR protects against hypercalcaemia probably by inhibiting bone resorption activity^(69)^. In cases of high concentrations of Ca in the blood, the hormone calcitonin that is secreted from the thyroid gland^(70)^ reduces serum Ca levels by decreasing osteoclastic bone resorption^(71,72)^ through stimulation of CALCR expression as demonstrated in rats^(73)^. Besides, rat osteoclast cells were identified as the cells expressing the highest amount of CALCR^(74,75)^, which is why the increase in CALCR mRNA expression in this study could reflect an increased number of osteoclasts. Increased extracellular P_i_ led to inhibition of RANK–RANKL-induced osteoclastic differentiation in the cell culture of a previous study^(76)^. In the present study, neither RANK nor RANKL gene expression changed in the caprine rib bone, although plasma P_i_ was diminished. OPG binds RANKL, thus preventing RANKL from binding to RANK and protecting against excessive bone resorption^(77)^. Therefore, the reduction in OPG mRNA abundance may indicate induced bone loss during P-reduction, as observed in glucocorticoid-induced bone loss in mice^(78)^. This lower OPG mRNA expression is induced by the reduction in FGF23 mRNA expression during P-restriction, which leads to an increase in PTH secretion^(79)^, and is reflected in a negative correlation of FGF23 mRNA with PTH1R mRNA in the present study. By binding to PTH1R on osteoblasts, PTH induces bone resorption through stimulation of RANKL expression and inhibition of OPG expression^(29)^.

This also supports the assumption that the elevated plasma Ca concentration was caused by bone resorption. Although RANKL gene expression did not change with P-restriction in this study, a positive correlation of PTH1R mRNA with RANKL mRNA (Fig. 2) was observed, supporting the hypothesis that bone resorbing activity increased. RANKL/OPG ratio shows the physiological ratio of bone formation and turnover increased with P-restriction in the present study. The higher this ratio, the more pronounced the bone resorption^(80)^.

Although neither RANK nor OPG gene expression nor the RANKL/OPG ratio changed in the goats during N-reduction, the increase in RANKL mRNA indicates an increase in osteoclast activation^(81)^, and this is supported by the previously mentioned rise in CTX concentration in the blood of N-reduced goats. Furthermore, the influence of PTH1R on bone resorbing activity in the N-restricted-fed goats was demonstrated by a positive correlation of PTH1R mRNA with RANKL mRNA expression (Fig. 2) and with CTX in blood.

In monogastric species, a reduced blood P_i_ concentration led to reduced synthesis of FGF23 from the bone due to restricted P-intake^(27)^. This effect was also demonstrated in ruminants in a previous study on P-depleted sheep^(82)^ as well as in the young goats of the present study receiving a P-reduction. The positive correlation of FGF23 mRNA with the blood concentration of P_i_ confirms the linear relationship. However, it is unclear which mechanisms the P_i_ regulates in FGF23 synthesis and secretion. In rat osteoblasts, an increase in PiT1 mRNA expression with reduced P_i_ was observed^(83)^, as was the case in the P-restricted goats of this study, suggesting that PiT1 plays an essential role in P_i_ transport activity in bone in addition to PiT2.

Furthermore, in mice, FGF23 expression in bone was lowered by reduced blood concentration of calcitriol via reduced VDR expression^(84)^. As mentioned above, reduced CYP27B1 gene expression in the kidney^(51)^ could be another reason for the reduction in FGF23 mRNA expression.

One signalling pathway that inhibits the expression of FGF23 in bone is the PI3K/Akt pathway. It is induced by insulin and IGF1 via their receptors’ INSR and IGF1R. Activation of the PI3K/Akt signalling pathway inhibits the transcription factor FOXO1 through phosphorylation, which leads to the down-regulation of FGF23 gene expression^(85)^. The increased expression of INSR, IGF1R, Akt1 and Akt2 in bone indicates activation of the PI3K/Akt signalling pathway by stimulated phosphorylation of FOXO1 during P-reduction in young goats, which resulted in lowered FGF23 mRNA expression. The positive correlation of INSR mRNA expression with Akt1 (Fig. 1(a)) and Akt2 (Fig. 1(b)) mRNA expression in this study indicates that an increase in INSR mRNA upregulates the expression of Akt1 and Akt2 mRNA in goats.

One pathway activating FGF23 expression is that of the JAK2/STAT3 signalling pathway. It is activated by leptin and GH through their receptors’ LEPR and GHR in bone^(25,26)^. During P-restriction, it cannot be excluded that the increased LEPR expression and GHR expression could induce the JAK2/STAT3 signalling pathway despite lowered FGF23 mRNA. In a study on mice, leptin was found to reduce the expression of CYP27B1 in the kidney. This reduction was mediated by LEPR in the murine renal proximal tubules to normalise elevated serum Ca and P_i_ concentrations^(23)^.

In summary, the prominent lower blood P_i_ concentration in the P-restricted goats reduced FGF23 mRNA expression in bone. Furthermore, an arrest of immature to mature osteoblastic differentiation was hypothesised during P-reduction indicating reduced bone formation. The potential reduction in bone formation was also reflected in a lower bone formation marker OC as well as a lower bone mineral content and bone mineral density. At the same time, higher OPG mRNA expression with P-restriction may indicate less inhibition of RANKL–RANK binding. Consequently, osteoclastic differentiation may increase which may result in bone resorption due to the lower P_i_ blood concentration. The potential increase in bone resorption was also reflected by an increase in the RANKL/OPG ratio. The increase in PTH1R mRNA expression with P-restriction may also reflect osteoblastic bone resorption. The increase in CALCR mRNA expression due to the high Ca concentrations in the P-restricted goats suggests an increase in osteoclast numbers, supporting the hypothesis of increased bone resorption. With N-restriction, blood Ca concentration was lower, which probably led to increased PTH1R gene expression in bone. This could reflect an increase in bone formation, which in turn might have stimulated bone resorption to release Ca from the bone. The increase in RANKL gene expression suggests the activation of osteoclasts. This hypothesised increase in bone turnover was also reflected in an increase in OC and CTX in the blood.

In addition to the molecular changes observed, it is important to address potential confounding factors that may have influenced our results. Future studies using pair feeding or more controlled feeding conditions are needed to differentiate the direct effects of treatment from those of reduced DM intake. IGF1 is a critical mediator in bone metabolism, influenced by nutrient intake including DM intake. The relationship between DM intake and IGF1 levels could confound the interpretation of the effects of N- and P-restrictions on bone health. This approach would help to delineate the specific contributions of nutrient restrictions *v*. overall dietary intake on bone health in young goats.

In conclusion, while our study highlights the critical roles of P and N in bone metabolism and the molecular mechanisms involved, it is essential to consider the potential confounding effects of DM intake and the role of IGF1 and other blood markers. Addressing these factors in future research through controlled feeding conditions will enhance our understanding of nutrient regulation of bone health and help to develop more effective dietary strategies for maintaining bone integrity in livestock.
